# COVID-19 in patients with paracoccidioidomycosis

**DOI:** 10.1371/journal.pntd.0011322

**Published:** 2023-05-08

**Authors:** Priscila Marques de Macedo, Lorena Macedo Pestana Benko, Eduardo Mastrangelo Marinho Falcão, Joshua D. Nosanchuk, Rodrigo Almeida-Paes, Antonio Carlos Francesconi do Valle

**Affiliations:** 1 Laboratory of Clinical Research on Infectious Dermatology, Evandro Chagas National Institute of Infectious Diseases, Oswaldo Cruz Foundation (Fiocruz), Rio de Janeiro, Brazil; 2 Departments of Medicine and Microbiology & Immunology, Albert Einstein College of Medicine, Bronx, New York, United States of America; 3 Mycology Laboratory, Evandro Chagas National Institute of Infectious Diseases, Oswaldo Cruz Foundation (Fiocruz), Rio de Janeiro, Brazil; University of Khartoum, SUDAN

## Abstract

**Introduction:**

In 2020, we reported the first patient with concomitant COVID-19 and paracoccidioidomycosis (PCM). Since then, no other cases have been recorded in the literature. We aim to update information on the occurrence of COVID-19 in patients with PCM followed at a reference center for infectious diseases at Rio de Janeiro, Brazil.

**Methods:**

We reviewed the medical records from patients diagnosed with PCM who presented with clinical symptoms, radiological findings, and/or laboratory diagnosis of COVID-19 at any time during their acute or follow-up care. The clinical profiles of these patients were described.

**Results:**

Between March 2020 and September 2022, we identified six individuals with COVID-19 among the 117 patients with PCM evaluated. The median age was 38 years and the male to female ratio 2:1. Most patients (n = 5) presented for evaluation due to acute PCM. The severity of COVID-19 ranged from mild to severe in acute PCM and only the single patient with chronic PCM died.

**Conclusions:**

There is a range of disease severity in COVID-19 and PCM co-infection and concomitant disease may represent a severe association, especially in the chronic type of the mycosis with pulmonary involvement. As COVID-19 and chronic PCM share similar clinical aspects and PCM is neglected, it is probable that COVID-19 has been hampering simultaneous PCM diagnosis, which can explain the absence of new co-infection reports. With the continued persistence of COVID-19 globally, these findings further suggest that more attention by providers is necessary to identify co-infections with *Paracoccidioides*.

## Author summary

Paracoccidioidomycosis (PCM) is a severe invasive mycosis, affecting the lungs and other important organs of patients in Latin America, notably Brazil. *Paracoccidioides* is the causative agent of PCM and it was recently included in the World Health Organization (WHO) fungal priority pathogens list to guide research, development and public health action. Since the beginning of COVID-19 pandemic, only one case of concomitant PCM and COVID-19 has been reported. Our work aims to investigate the occurrence of COVID-19 in patients with PCM followed at a reference center for infectious diseases in a Brazilian endemic region for PCM. Between March 2020 and September 2022, we identified six individuals with COVID-19 among the 117 patients with PCM evaluated. Although we found a high range of disease severity in the cases analyzed, concomitant disease also represented a severe association. As COVID-19 and PCM may share similar clinical aspects and PCM is neglected, it is probable that COVID-19 has been hampering simultaneous PCM diagnosis, which can explain the absence of new co-infection reports. Our findings call for more attention by health professionals to identify co-infections with *Paracoccidioides*.

## Introduction

Paracoccidioidomycosis (PCM) is a severe systemic mycosis, endemic in Latin America, mainly in Brazil. The infection typically occurs after inhalation of infective fungal propagules in the course of activities involving soil management and may progress acutely or chronically to cause clinical disease, chronic manifestations being most common, especially in rural workers. The lungs are the most affected organs in chronic PCM, while the mononuclear phagocyte system is involved in the acute “juvenile” cases of this mycosis [1]. Rio de Janeiro state is an important endemic region for this mycosis, and there has recently been an increase in the incidence of acute PCM forms in urban areas [2].

When COVID-19 emerged in Brazil in late February 2020 [3], we expected to observe a clinical impact in individuals with PCM, especially in patients with the chronic presentation of the mycosis with pulmonary impairment. On the other hand, COVID-19 shares similar clinical signs with endemic pulmonary mycoses, which can hamper the diagnosis of neglected fungal diseases [4]. These overlapping clinical signs are mostly related to the pulmonary and upper airway involvement of COVID-19 and chronic PCM, both leading to respiratory symptoms such as cough, hoarseness, and dyspnea, while acute PCM rarely affects these organs. In 2021, Heaney et al. [5] reviewed the interaction between COVID-19 and coccidioidomycosis, analyzing the risk factors and the implications of this co-infection, and highlighted how the COVID-19 pandemic might exacerbate delays in the diagnosis of the mycosis. Similarly, the difficulty of diagnosing PCM during hospitalization through the COVID-19 pandemic has also been reported, resulting in delayed diagnosis of PCM [6]. The confounders of overlapping clinical manifestations may explain in part the absence of new case reports of PCM and COVID-19 co-infection.

In 2020, we reported the first case of COVID-19 and PCM co-infection in a young male patient, and discussed the clinical profile and severity of this association [7]. Since this first report, no other cases of this co-infection have been recorded in the literature to date. Given the lack of additional publications and information about co-infections with PCM and COVID-19, we sought to characterize the occurrence and presentations of COVID-19 in a cohort of patients with PCM at a reference center for infectious diseases in a PCM endemic region.

## Methods

### Ethics statement

The Research Ethics Committee of INI/Fiocruz approved this study (appreciation numbers: 18524919.1.0000.5262 and 26066619.0.0000.5262). A written consent form was obtained from the patients included in the study. The patients’ data were anonymized/de-identified to protect patients’ privacy/confidentiality.

### Study design

We performed a review of medical records from patients with PCM followed at the Evandro Chagas National Institute of Infectious Diseases (INI/Fiocruz) between March 2020 and September 2022, who presented clinical, radiological, and/or laboratory findings of COVID-19 during their care, from initial diagnosis to the post treatment phase.

### Inclusion criteria

We defined the following criteria for COVID-19 diagnosis: (a) suspected case: presence of clinical symptoms, notably fever and/or respiratory symptoms along with a potential exposure to SARS-CoV-2 through a close contact with a confirmed or suspected case; (b) probable case: suspected case with typical radiological findings of COVID-19; (c) confirmed case: suspected case with a positive antigen or molecular test against SARS-CoV-2 performed in the healthcare facilities, by trained professionals. The PCM diagnosis was based on the recommendations of the Brazilian Guidelines for the clinical management of PCM [1]. Briefly, (a) suspected case: presence of clinical manifestations of PCM, excluding tuberculosis and other differential diagnosis, for at least four weeks; (b) probable case: suspected case presenting anti- *Paracoccidioides* serum antibody titers detected by quantitative double immunodiffusion test; (c) confirmed case: presence of fungal elements suggestive of *Paracoccidioides* spp. in direct examination, histopathology or culture [1]. All PCM patients were routinely screened for tuberculosis (TB) including smear microscopy (Ziehl-Neelsen stain), culture (Lowenstein-Jensen method), and Xpert MTB/RIF from clinical specimens. The clinical aspects and possible risk factors for PCM and COVID-19 co-infection are further discussed.

## Results

**Fig 1:**
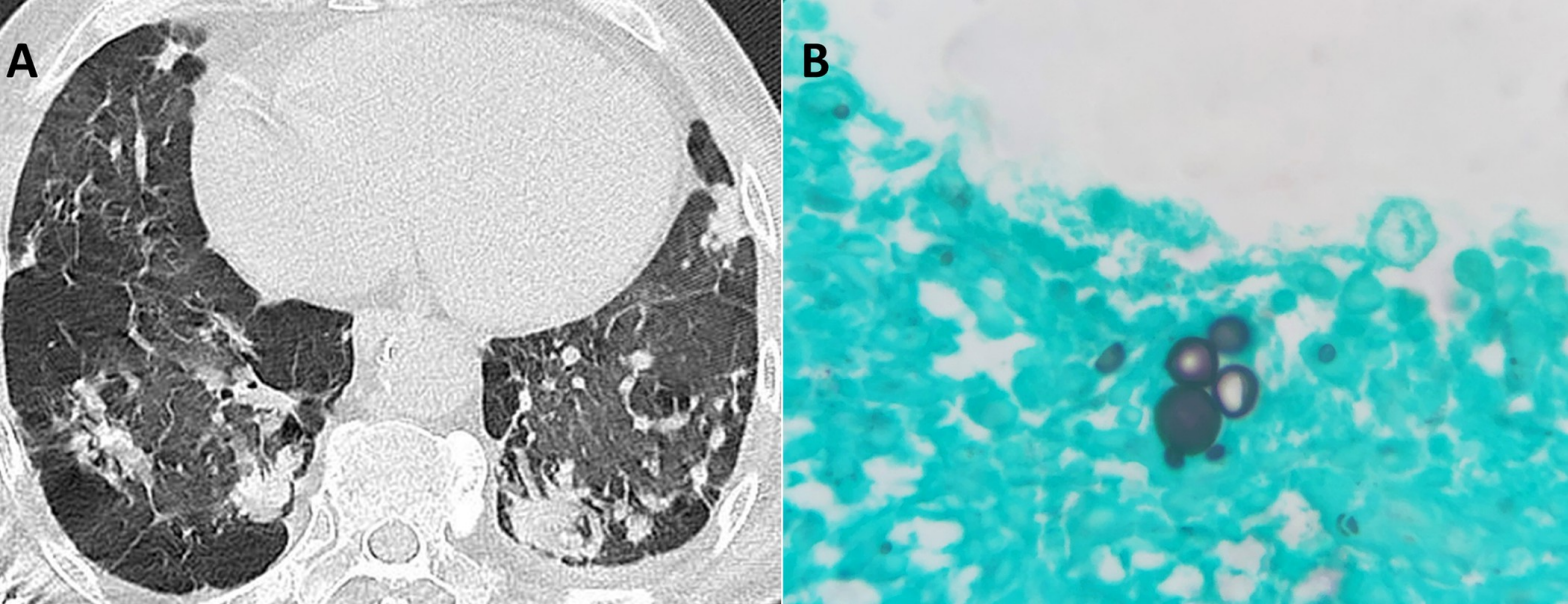
(A) Chest tomography from a patient included in this study (case 2) showing peripheral and coalescent ground-glass opacities better seen in the right lung. This 57-year-old male patient was infected by *Paracoccidioides* sp. in the past, while developing construction activities during 20 years. He was a heavy smoker, developed chronic paracoccidioidomycosis (PCM), and was admitted at our institution presenting a 9-month painful tongue lesion, evolving with emaciation, cervical lymph nodes enlargement, and lung disease. PCM was diagnosed through histopathology from biopsies of the oral lesion and cervical lymph node, which revealed a chronic granulomatous inflammatory process along with multiple budding cells compatible with *Paracoccidioides* spp. yeast-like cells, better seen in the Grocott’s methenamine silver stain (B). The patient was treated with itraconazole and trimethoprim/sulfamethoxazole for 35 months due to poor adherence. When COVID-19 infection occurred, this patient was under the post-therapeutic follow-up, and presented lung sequelae, such as severe pulmonary fibrosis and chronic obstructive pulmonary disease (COPD). His COVID-19 disease was severe, requiring invasive ventilation, and he died four days after hospitalization.

In the period of study, we identified six patients with COVID-19 among 117 patients evaluated PCM, including the patient we previously reported [7]. Most of them (88.3%) came from *Baixada Fluminense*, an urban region with low socioeconomic conditions, where recently an increase in acute PCM case numbers was documented [2]. Four patients had laboratory confirmed COVID-19, one had a negative test but presented typical radiographic findings of COVID-19 (Fig 1), and one, although not tested, had flu-like symptoms after a close contact with a suspected case, but was no longer symptomatic at the time of our routine consultation. Table 1 depicts the main clinical characteristics of the patients included in this study.

**Table 1:** Summary description of the cohort of patients with paracoccidioidomycosis and COVID-19 co-infection.

Case	Age	Sex	PCM form	PCM diagnosis	PCM follow-up	PCM sequelae	COVID diagnosis	COVID severity	Outcome
**1**	19	M	Acute	Direct examination and culture from lymph node aspirate	During treatment (AMB)	Lymph stasis	RT-PCR NF swab (C)	Severe	LFU
**2**	63	M	Chronic	Histopathology of tongue lesion and lymph node	Post treatment	Pulmonary fibrosis	Radiological pattern ^#^ (P)	Severe	Death
**3**	42	M	Acute	Direct examination and culture from lymph node aspirate	During treatment (ITZ)	Cutaneous scars	RT-PCR NF swab (C)	Mild	Cure
**4**	46	M	Acute	Direct examination and culture from lymph node aspirate	Post treatment	Hepatic fibrosis	RT-PCR NF swab (C)	Moderate	Cure
**5**	34	F	Acute	Direct examination from lymph node aspirate	Post treatment	Lacrimal obstruction	LFA (C)	Mild	Cure
**6**	36	F	Acute	Histopathology of lymph node	Post treatment	Tracheostomy	Flu-like symptoms (S)	Mild	Cure

PCM: paracoccidioidomycosis; PCM follow-up: the moment of the PCM care when the patient presented clinical manifestations of COVID-19; COVID-19 severity: based on the WHO clinical progression scale [8]; M: male; F: female; AMB: amphotericin B; ITZ: itraconazole; RT-PCR: Reverse Transcription Polymerase Chain Reaction; NF: nasopharyngeal; # Radiological pattern with typical findings of COVID-19 (ground glass opacities [GGOs] and consolidation with bilateral and peripheral distribution); LFA: antigen test by lateral flow assay; (C) confirmed, (P) probable, or (S) suspected case; LFU: lost of follow-up. Case 1 has been previously reported [7].

Three patients presented with mild symptoms of COVID-19, including low-grade fever, headache, and other common cold symptoms (cough, sneezing and nasal discharge). The other three patients required hospitalization due to hypoxia. One patient was considered to have moderately severe disease and was treated with high-flow nasal oxygen, corticosteroids, and favipravir. He had 13 days of intensive care unit (ICU) stay, and did not develop additional complications of COVID-19. Both patients with severe COVID-19 required invasive mechanical ventilation. One patient had acute PCM under treatment with pulmonary involvement and we previously reported this case [7]. At the time that we published the description of his case (August 2020) this patient was still hospitalized. Although recovered from acute disease, he subsequently developed a dilated cardiomyopathy as a complication of COVID-19 after discharge, but recovered and was lost follow-up after 17 months of PCM treatment. The other patient with severe COVID-19 had chronic PCM under post-therapeutic follow-up with severe pulmonary fibrosis, chronic obstructive pulmonary disease (COPD), and, despite treatment in the ICU with mechanical ventilation, corticosteroids, and antibiotics, died four days after admission due to rapid decompensation due to complications of COVID-19. Although only two patients were under treatment for PCM when they had COVID-19, there was no need to change the therapeutic regimen for PCM in these cases. None of the patients from this study presented TB co-infection.

## Discussion

Invasive fungal infections have been an important complication of COVID-19 infection especially in severe disease, with a significant incidence of mucormycosis, aspergillosis and candidiasis [9–11]. Due to these complications, consensus guidelines for the management of certain mycoses in the setting of SARS-CoV-2 have been established [12]. Concurrent COVID-19 and endemic systemic mycoses have been well documented and a recent review indicates the occurrence of this condition worldwide [13]. In this review, the authors identified seventy-eight patients with COVID-19 associated with an endemic systemic mycosis. Coccidioidomycosis was most frequently reported, followed by histoplasmosis, with cases occurring mostly in the Americas. Blastomycosis and PCM accounted for one case each, in the U.S and Brazil, respectively. The Brazilian case was initially published by our group and was also herein updated to report his final outcome, which was not made in the previous publication [7]. To date, emergomycosis and talaromycosis associated with COVID-19 have not been reported. It has been noted that the immune dysregulation and inflammation occurring in COVID-19 likely leads to a higher risk of symptomatic disease and reactivation of endemic mycoses in individuals with previously latent infections [12]. In addition, the use of corticosteroids to treat severe COVID-19 induced an immunosuppressive condition that would contribute to reactivation.

In our patient populations, we did not observe an increase in PCM cases following COVID-19 as reported for other endemic mycoses [14]. Therefore, the immune alterations induced by this viral disease or its treatment does not seem to promote a reactivation of latent infection due to *Paracoccidioides* spp. or to exacerbate PCM.

Five of the six patients herein studied had acute PCM, without prior comorbidities. At present, it is unclear whether this finding is due to the new epidemiological profile of PCM in the state of Rio de Janeiro characterized by a higher incidence of acute cases [2] or whether this is related to an increased susceptibility of the immune response of acute PCM against SARS-CoV-2. Among these acute PCM cases, mild COVID-19 prevailed, which is consistent with the scarcity of lung impairment in acute PCM, although it could also be due to a control of PCM disease at the time of the COVID-19 diagnosis in these mild cases. In fact, the patient with acute PCM who developed severe COVID-19 had severe active PCM with pulmonary involvement (pleural effusion) [7]. On the other hand, the death of the single patient with severe COVID-19 and a history of chronic PCM, but with pulmonary sequelae and COPD, was attributed to the infection with SARS-CoV-2. Therefore, acute or chronic lung involvement in PCM may contribute to a worse outcome of COVID-19.

It is noteworthy that itraconazole, which is currently the first line oral antifungal drug to treat PCM, has *in vitro* activity against SARS-CoV-2 [15–17]. Although this antifungal drug has not been proven to be effective for COVID-19 treatment [16], PCM patients receiving itraconazole would have experienced a protective effect of this drug against SARS-CoV-2, by reducing viral replication rates. Additionally, at least 14 non-antifungal drugs with potential activity against SARS-CoV-2 have shown *in vitro* antifungal activity against *P*. *brasiliensis* [18]. Therefore, this may also have contributed to protecting individuals against disease due to *Paracoccidioides* spp., which may perhaps explain, in part, the absence of post-COVID-19 PCM cases. The patients from this study did not receive any drugs with known clinical activity against SARS-CoV-2, except favipravir, which does not have antifungal activity against *Paracoccidioides* spp. [18]

Our findings suggest that co-infection with SARS-CoV-2 and *Paracoccidioides* manifests in mild to lethal outcomes with increased odds of poor outcomes when the lungs are compromised due to PCM. As COVID-19 and chronic PCM share similar clinical aspects and this mycosis is neglected, it is likely that COVID-19 has been hampering PCM diagnosis, which can explain the absence of reports of new co-infected cases in regions endemic for *Paracoccidioides* sp. In addition, according to the authors’ personal experience, acute PCM cases continue to be misdiagnosed as other diseases such as tuberculosis and lymphoma. Therefore, clinicians must be aware of this possible association of increased COVID-19 severity in PCM endemic areas. The research advances of new therapeutic options for COVID-19 and studies repurposing drugs to both PCM and COVID-19 may help to understand the epidemiology of this co-infection, as well as to promote advances in the therapeutic management of both diseases. Notably, mild COVID-19 cases, especially after vaccination, can account for the lack of co-infection reports. Regardless, more studies are needed for a better comprehension of this important issue, and mandatory reporting of PCM should be a public health priority.
